# Characterisation and Immunogenicity of *Neisseria cinerea* outer membrane vesicles displaying NadA, NHBA and fHbp from *Neisseria meningitidis* serogroup B

**DOI:** 10.3389/fimmu.2024.1473064

**Published:** 2024-09-24

**Authors:** Shathviga Manoharan, Theo A. Farman, Stavroula Piliou, Pietro Mastroeni

**Affiliations:** Department of Veterinary Medicine, University of Cambridge, Cambridge, United Kingdom

**Keywords:** modified outer membrane vesicles, mOMVs, *Neisseria cinerea*, meningitidis, fHbp, NHBA, NadA, *Neisseria meningitidis*

## Abstract

More affordable and effective vaccines against bacterial meningitis caused by *Neisseria meningitidis* serogroup B are still required for global prevention. We have previously shown that modified outer membrane vesicles (mOMVs) from commensal *Neisseria cinerea* can be used as a platform to induce immune responses against meningococcal antigens. The aim of the present study was to use a combination of two genetically engineered mOMVs to express multiple antigens from *N. meningitidis* known to be involved in protective immunity to meningococcal meningitis (different variants of factor H binding protein (fHbp), *Neisseria* Heparin Binding Antigen (NHBA) and *Neisseria* Adhesin A (NadA)). Antigen expression in the mOMVs was confirmed by Western blotting; detoxification of the lipooligosaccharide (LOS) was confirmed by measuring human Toll-like receptor 4 (hTLR4) activation using *in vitro* cell assays. Mice immunised with a combination of two mOMVs expressing fHbp, NHBA and NadA produced antibodies to all the antigens. Furthermore, serum bactericidal activity (SBA) was induced by the immunisation, with mOMVs expressing NadA displaying high SBA titres against a *nadA^+^* MenB strain. The work highlights the potential of mOMVs from *N. cinerea* to induce functional immune responses against multiple antigens involved in the protective immune response to meningococcal disease.

## Introduction

*Neisseria meningitidis* is a Gram-negative diplococcus that asymptomatically colonises the nasopharyngeal mucosa of healthy carriers. On some occasions, the bacterium traverses the mucosal epithelium and invades the bloodstream, leading to invasive meningococcal disease (IMD), a potentially life-threatening disease and a major cause of bacterial meningitis and septicaemia worldwide (1). There are 13 serogroups of *N. meningitidis* based on the composition of polysaccharide capsule, with most IMD cases primarily caused by serogroups A, B, C, W, Y and X (2).

Although conjugated polysaccharide vaccines are used for immunization against *N. meningitidis* serogroups A, C, Y, and W (2), this has not been the case for *N. meningitidis* serogroup B (MenB). A polysaccharide-based vaccine for MenB was disregarded for two major reasons: its theoretical risk for autoimmunity due to the similarity of the chemical composition to sialic acid found on many human cell types and its low immunogenicity (3, 4). Therefore, several non-capsular outer membrane vesicle (OMV) vaccines were used during epidemics of MenB (5), and vaccines based on recombinant proteins identified by proteomic and reverse vaccinology approaches are currently available (6, 7).

Two protein-based vaccines against MenB currently in use are the four-component 4CMenB vaccine (Bexsero^®^, GlaxoSmithKline) and the bivalent fHbp2086 vaccine (Trumenba^®^, Pfizer). These vaccines use subcapsular proteins that are widely present not only in serogroup B strains, but also across different meningococcus serogroups (8, 9). The factor H binding protein (fHbp) vaccine (formally known as LP2086) consists of two lipidated fHbp (8). The 4CMenB vaccine contains three components that were identified by reverse vaccinology based on the complete genome sequence of a pathogenic reference MenB strain (strain MC58): the fHbp variant 1.1 fused to the genome-derived *Neisseria* antigen (GNA) 2091, the *Neisseria* adhesin A variant 2/3 peptide 8 (NadA-8), and the *Neisseria* Heparin binding antigen peptide 2 (NHBA-2) fused to GNA1030 (9). Besides these three recombinant surface-exposed protein antigens, the vaccine contains detergent-extracted OMVs from the New Zealand strain NZ98/254 containing porin A (PorA) P1.4 (10). While these vaccines have successfully decreased the incidence of group B meningitis in both infants and adults, the challenge of high production costs persists (11).

FHbp is a surface-exposed protein expressed in almost all *N. meningitidis* strains and divided in two subfamilies, A and B (12). Due to the variations in amino acid sequences, each fHbp protein can be identified by an ID number (12). FHbp binds to human factor H, a negative regulator of the alternative pathway of complement activation and is an immune-evasion factor in *Neisseria* (13, 14). NHBA is a surface protein that binds to heparin, which can confer a level of serum resistance as well as binding to heparin sulphate proteoglycans to aid in bacterial adherence to epithelium (15, 16). An additional role of NHBA is to promote biofilm development through the binding with extracellular DNA involved in the initial stages of cell-to-cell attachment (17). NHBA is present in all *N. meningitidis, N. gonorrhoeae* and some commensal *Neisseria* species (18). NadA is an outer membrane protein (OMP) that promotes adhesion to and invasion of host cells during bacterial infection and colonisation of the human respiratory tract (18). There are four variants of NadA, with variants 1 and 2/3 found in invasive meningococci (19). Unlike fHbp and NHBA, the *nadA* gene is only found in approximately 30% of *N. meningitidis* isolates, but importantly it is present in approximately 75% of hypervirulent MenB strains (18, 20, 21).

*In vitro* measurement of functional antibodies has been considered for decades as a correlate of protection against many bacterial species (22). Serum bactericidal activity (SBA) assays are commonly used as correlates of protection for vaccines against *N. meningitidis* (23–26), with SBA titres having been used to prioritise antigens for incorporation into the Bexsero^®^ vaccine.

OMVs are an attractive vaccine platform for their excellent adjuvanticity (27, 28), the possibility of modifying them to include surface-expressed heterologous antigens (29–32), and the simplicity of their production and purification process (33). Indeed, OMV-based vaccines against bacterial pathogens have already reached the market (34, 35), while others are in advanced clinical phases (36, 37). Vaccines developed against MenB based on OMVs include VA-MENGOC-BC [Finlay Institute] used in Cuba, MenBvac [Norwegian Institute of Public Health] in Norway, and MeNZB [Novartis] in New Zealand. However, as they were designed for specific strains, this results in poor coverage across the diverse range of MenB strains.

Our group has previously shown the immunogenicity of genetically modified OMVs (mOMVs) from the commensal BSL-1 *Neisseria cinerea* with key antigens such as NHBA and fHbp being expressed (32). OMVs purified from the commensal biosafety level 1 (BSL-1) *Neisseria lactamica* have also been studied as a potential vaccine against *N. meningitidis* (38–40). The use of a non-pathogenic *Neisseria* strain has enormous benefits in terms of ease and safety of manipulation in the laboratory, ease of production, and low production costs. OMVs from commensal *Neisseria* spp. that are phylogenetically placed near *N. meningitidis* also have the potential to induce immune responses against antigens with a high level of homology (and therefore potential immunological cross-reactivity) between *Neisseria* species (32, 39).

In the present study, we capitalised on our previous proof-of-principle work (32) on the use of *N. cinerea* OMV. In the current study we have leapt towards the delivery of a combination of several *Neisseria* antigens known to be involved in the protective response against meningococcal meningitis. We have used the commensal strain *N. cinerea* ATCC^®^ 14685™ that naturally expresses fHbp_ID100_ from subfamily B, with 97% aa identity to the fHbp present in Bexsero^®^ (32, 41). Along with fHbp_ID45_ from subfamily A and NHBA peptide 2 (NHBA-2, included in Bexsero^®^), we have also incorporated NadA variant 2/3 peptide 8 (NadA-8, included in Bexsero^®^) into the mOMVs to provide a broader coverage of MenB strains. Antigen combinations of fHbp, NHBA and NadA are known to play a key role in the protective activity of the currently licensed vaccines against MenB (9). We immunised mice with two different vesicles incorporating the antigens listed above and tested antibody responses by ELISA and Western blotting (WB), as well as measuring SBA as a correlate of protection.

## Materials and methods

### Bacterial strains and growth conditions

All commensal *Neisseria* strains (**Supplementary Table S1**) were grown on gonococcal (GC) base medium agar plates (Oxoid) with Kellogg’s supplements (42) or in GC liquid broth (Oxoid) with Kellogg’s supplements and 0.042% (w/v) sodium bicarbonate at 37C, 5% CO_2_. N*. meningitidis* MC58 was grown on horse blood agar plates at 37C, 5% CO_2_. Erythromycin (10 μg/mL), gentamicin (10 μg/mL), kanamycin (40 μg/mL) and chloramphenicol (2.5 μg/mL) were added to GC agar or GC broth as required for selective growth conditions.

### Generation of genetically modified *N. cinerea* ATCC^®^ 14685™ strains

Briefly, all the insertions and deletions of genes into the chromosome of *N. cinerea* ATCC^®^ 14685™ were performed using constructs synthesised by GenScript. The constructs were inserted into pUC18 vector (**Supplementary Table S2**), linearised with the restriction enzyme HindIII (NEB) and purified using the Monarch PCR and DNA Cleanup Kit (NEB). The fragments were amplified with Q5 Hot Start High Fidelity 2X Master Mix (NEB) by PCR with the appropriate primer pairs (**Supplementary Table S3**). The resulting fragments were purified using the Monarch PCR and DNA Cleanup Kit (NEB), confirmed by Sanger Sequencing (Source Biosciences) and transformed into the appropriate *N. cinerea* ATCC^®^ 14685™ strains (**Supplementary Table S1**).

### mOMV production

For production of mOMVs, bacteria were streaked onto GC agar plates containing the appropriate antibiotics and incubated at 37°C, 5% CO_2_ for 16-48 hours. Colonies were picked and used to inoculate 5 mL of GC broth to obtain an optical density (OD) at 600 nm (OD_600_) of ~0.1, and then incubated at 37°C, 5% CO_2_, shaking at 150 rpm. Once the culture reached OD_600_ of ~1.0, 5 mL of the broth was used to inoculate 50 mL of fresh, pre-warmed GC broth (1:10 dilution) and incubated at 37°C, 5% CO_2_, shaking at 150 rpm. When the culture reached OD_600_ of ~1.0 (typically 3-4 hours later) and the OD_600_ value remained constant or started to decrease after a further hour, the culture was removed from incubation and the bacteria were separated from the media by centrifuging at 2,200 x g, 4°C for 40 minutes. The supernatant was then filtered with a 0.22 μm polyethersulfone membrane (Millipore). To check for absence of bacterial growth, 10 μL of supernatant was streaked on a GC agar plate and incubated overnight at 37°C, 5% CO_2_.

To isolate the mOMVs that were blebbed during culturing, the supernatant was ultracentrifuged at 45,000 rpm at 4°C for 2 hours using a Beckman Coulter L-80 Ultracentrifuge (Type 50.2 Ti rotor), before the pellet was resuspended in 1X PBS. Afterwards, the suspension was spun again, and the pellet was allowed to resuspend in 100-200 μL of fresh 1X PBS overnight, before finally being spun through 0.22 μm spin filters (Corning). The resulting OMV sample was then stored at -20°C until further analysis.

### Recombinant proteins, monoclonal and polyclonal antibodies

Recombinant proteins fHbp_ID100_ (rID100), fHbp_ID45_ (rID45), NHBA-2 (rNHBA-2) and NadA-8 (rNadA-8) were custom-made by GenScript. Briefly, genes expressing fHbp_ID100_, fHbp_ID45_, NHBA-2 and NadA-8 were cloned into the pET30a vector using the NdeI/HindIII cloning sites. A C-terminal His-tag was used for *fHbp* genes, while an N-terminal His-tag was used for *nhba-2* and *nadA-8* genes. The signal peptide sequence was removed from the N-terminal end of NHBA-2 and NadA-8. rID100 was purified by nickel chromatography and size exclusion chromatography while rID45, rNHBA-2 and rNadA-8 were purified by nickel chromatography. For plasmid generation, competent *Escherichia coli* TOP10 was used, while for protein expression *E. coli* BL21 StarTM (DE3) was used.

The commercially available monoclonal antibodies JAR5 and JAR13 were purchased from NIBSC and Merck, respectively. JAR5 is specific for fHbp subfamily B and JAR13 is specific for fHbp subfamily A (43). To generate polyclonal antibodies against NHBA-2 and NadA-8, the purified recombinant proteins were used to immunise rabbits in three doses (carried out by Genscript, see **Supplementary Table S4**).

### Confirmation of mOMV antigenic profile

The Pierce™ bicinchoninic acid (BCA) Protein Assay Kit (ThermoFisher) was used to determine the total protein concentration of purified mOMVs in relation to a standard curve obtained with commercially available bovine serum albumin (BSA) and followed the manufacturer’s instruction.

To determine expression of antigens in OMV preparations, SDS-PAGE was used to separate the individual proteins by molecular weight. For each preparation, 1.5 μg of OMVs was mixed with 2X sample buffer (NuPage Sample Reducing Agent (10X) and NuPAGE LDS Sample Buffer (4X) in MilliQ water) and heated at 95°C for 20 minutes. Recombinant proteins (rID100, rID45, rNHBA2 and rNadA) were also prepared as a positive control to 0.1 μg in 1X sample buffer and heated at 95°C for 20 minutes. Controls and samples were then loaded on either NuPAGE Novex 12% Bis-Tris Protein Gels (Thermo Scientific), using NuPAGE MOPS SDS Running Buffer (Thermo Scientific) to check fHbp expression or NuPAGE 7% Tris-Acetate Protein Gels (Thermo Scientific) using NuPAGE Tris-Acetate Running Buffer (ThermoFisher) to check NHBA and NadA expression. The gels were also loaded with 10-250 kDa Colour Prestained Protein Standard Ladder (NEB). NuPAGE Novex 12% Bis-Tris Protein Gels were then ran at 100 V for 25 minutes and then 200 V for 40 minutes, whilst NuPAGE 7% Tris-Acetate Protein Gels were ran at 80 V for 25 minutes followed by 120 V for 40 minutes.

Subsequently, gels were used for WB. The Bio-Rad Turbo Transfer System was used to transfer the separated proteins from the gels to a nitrocellulose membrane using a ‘Mixed Molecular Weight’ program (25 V for 7 minutes). All membranes were then blocked with 10% (w/v) milk in 1X PBS for 2 hours at room temperature on a rocking platform, before being washed in wash buffer (0.05% Tween-20 in 1X PBS) six times. For incubation with a primary antibody solution, the relevant membranes were incubated overnight with the specific antibodies prepared as indicated in **Supplementary Table S4** in buffer solution (10% (w/v) milk, 0.05% Tween- 20 in 1X PBS). After another wash step, membranes were incubated for a further 2 hours with the relevant secondary antibody solution (See **Supplementary Table S4**). After a final wash, the membranes were developed using 1 mL BCIP^®^/NBT-Purple Liquid Substrate System (Sigma) and the reaction was then stopped by washing with MilliQ water before images were taken.

### Dynamic light scattering

Particle size distribution of mOMVs was evaluated by dynamic light scattering (DLS), with a Malvern Zetasizer Nano S containing a HE-Ne laser of 633 nm wavelength. mOMV samples were diluted to a 0.1 ug/uL dilution in PBS, then read at 25°C. Each sample was read in triplicates to measure distribution of OMVs, and the resulting readouts included average diameter and polydispersity index (PdI), using the Zetasizer 7.11 software (Malvern).

### *In vitro* toxicity assays with mOMVs, using HEK-blue cell lines

To determine the toxicity of mOMVs, *in vitro* human Toll-like receptor (hTLR4) stimulation assay was carried out using the commercially available HEK-Blue hTLR4 and HEK-Blue Null2 cells (InvivoGen). Cells were seeded and maintained as per manufacturer’s instruction (InvivoGen). In a 96-well flat bottom microplate, 20 μL of OMV sample were aliquoted from 1,000 ng/μL and serially diluted to 0.1 ng/μL. As a positive control, 20 μL of ultrapure lipopolysaccharide from *Escherichia coli* K12 (LPS-EK, InvivoGen) were aliquoted from 1,000 ng/μL and serially diluted to 0.1 ng/μL. OMV sample from *N. cinerea* ATCC^®^ 14685™ WT (OMV WT) was also used as a control to compare with mutant mOMVs. 80μL of either HEK-Blue cell suspension was added to the relevant wells and the plates were then incubated at 37°C in 5% CO2 for 24 hours. Subsequently, 100 μL of each supernatant was transferred to a new 96-well microplate and read using a microplate reader (Byonoy), at 620 nm (OD_620_). The ratio of SEAP induced colour change was then calculated: 


SEAP ratio=(Average sample OD620 ÷ Average blank well (buffer  only))

### Transmission electron microscopy

mOMVs were imaged by transmission electron microscopy (TEM), using a Tecnai G20 transmission electron microscope (FEI/ThermoFisher Scientific). Approximately 1 μg of each OMV preparation, diluted in 1X PBS, were place under a glow-discharged 400 mesh copper/carbon film grids (EM Resolutions) and left to be adsorbed for two minutes. The grids were then washed twice by passing over deionised water drops to remove buffer salts, before being passed on 2% (w/v) aqueous uranyl acetate to improve negative staining. After excess uranyl acetate was removed, the grids were then left to dry at room temperature. Images were then made at an accelerating voltage of 200 kV using a 20 μm objective aperture to improve contrast, using an ORCA HR CCD camera (Advanced Microscopy Techniques Corp, USA) for capture.

### Mouse immunogenicity studies

The study was performed at the University of Cambridge, under the appropriate Home Office licences and Ethical Review Committee approval. The dose formulations were prepared on the day of dosing, where the required concentration of relevant mOMV was added with Alhydrogel adjuvant (14 mg of Alhydrogel per 1 mg of total mOMVs) in 1X PBS and allowed to mix for one hour by rotation at room temperature. In each study groups of 6-8 weeks old female C57BL/6 mice were immunised intraperitoneally (IP) in three doses with 10 μg or 20 μg mOMVs (whole protein content), each ~21-28 days apart with control animals receiving Alhydrogel only (see **Table 1**).

**Table 1:** The different combinations of mOMVs injected in mice IP.

Group	Antigen	µg tot protein
**1)**	OMV ID100 NadA-8	10.00
**2)**	OMV ID45 NHBA-2	10.00
**3)**	OMV ID100 NadA-8	20.00
**4)**	OMV ID45 NHBA-2	20.00
**5)**	OMV ID100 NadA-8 + OMV ID45 NHBA-2	20.00
**6)**	Alhydrogel only	0.00

Blood samples were collected ~21 days after the third dose, by cardiac puncture under terminal anaesthesia and sera were stored at -20C until further use.

### Enzyme linked immunosorbent assay on mice sera

For the purpose of testing mice sera with indirect ELISAs, 96-well, flat bottomed, clear immunoassay plates (Thermo Scientific) were coated with 2 μg/mL of mOMVs or 0.25-2 μg/mL recombinant proteins and left at 4°C overnight. The plates were then washed six times in wash buffer (PBS-Tween-20 (v/v 0.05%) (PBS-T)), dried, and incubated at room temperature for 1 hour in 100 μL of blocking buffer (PBS-milk (w/v 5%)). Then, the plates were washed in wash buffer six more times, dried, before 2 hours of incubation with 50 μL of mouse sera diluted in PBS-T with 5% (w/v) milk in triplicate wells. For mOMV-coated plates, sera were diluted 1:27,000, then serially diluted, 1:3, three more times. For rID100, rID45 and rNHBA-2 coated plates, sera were diluted 1:20, then serially diluted, 1:2, three more times. For rNadA-8 coated plates, sera were diluted 1:2,000, then serially diluted, 1:2, three more times. Following a further washing step, wells were either incubated for 1 hour with 50 μL of anti-mouse IgG in PBS-T or anti-rabbit IgG in PBS-T (see **Supplementary Table S4**).

For development, plates were washed six times, and 100 μL of development solution was added (5x diethanolamine buffer (ThermoFisher) diluted in deionised water along with 1mg/mL 4-nitrophenylphosphate) and incubated at room temperature. The OD was read after 20 minutes of development time, using a microplate reader (Byonoy) at 405 nm (OD_405_), then again, every 5 minutes for a further 35 minutes. The OD at 405 nm (OD_405_) in each well was measured using a microplate reader (Byonoy) after 35 minutes for mOMV-coated plates or 45 minutes for mOMV-coated plates, ensuring readings represent the linear part of the curve. The average OD_405_ value was calculated between the technical triplicates, and the average ‘Blank’ OD_405_ reading was subtracted to yield the final titre value.

For data representation, the OD_405_ at 1:27,000 serum dilution was used for mOMV-coated plates, 1:40 serum dilution was used for plates coated with rID45 and rNHBA-2, 1:80 serum dilution was used for plates coated with rID100 and 1:4,000 serum dilution was used for plates coated with rNadA, as these dilutions are within the linear part of the curve.

JAR5, JAR13, and the rabbit polyclonal antibodies -NHBA-2, -NadA-8 were used as positive controls to ascertain reproducibility between different ELISA plates, at dilutions that fall in the linear part of predetermined OD_405_ *vs.* dilution curves (see **Supplementary Table S4** for concentration). Using these controls, the differences in OD_405_ between plates were <20%.

### Serum bactericidal activity assay

SBA assays were carried out by UK Health Security Agency, Vaccine Evaluation Unit (Manchester, UK), as described by Lucidarme et al. (44). *N*. *meningitidis* serogroup B strains 5/99 (Mar07) and M17 240102 were used for the assay. Pooled sera were heated before testing to deactivate the innate complement. For the assay, complement-preserved human serum was used. It should be noted that the lower limit of quantification for the SBA due to the specified stating dilution is 8. If any samples have SBA titres below 8, this will be reported as <8.

### Statistical analyses

Quantile-quantile plots have revealed that the ELISA data were not normally distributed and therefore distribution of ELISA data was analysed using the Kruskal-Wallis test with Dunn’s correction for multiple comparisons. hTLR4 stimulation assay was analysed using two-way ANOVA with Dunnett’s multiple comparisons test. All analyses were performed on GraphPad Prism Version 9.4.1.

## Results

### Expression of fHbp_ID100_ and NadA-8 in *N. cinerea* ATCC^®^ 14685™ mOMVs

While it has been observed from genome sequencing that some *N. cinerea* strains contain intact homologues of the *nadA* gene (45), *N. cinerea* ATCC^®^ 14685™ contains remnants of *nadA* variant 4/5 (see **Supplementary Results** and **Supplementary Figure S1-S3**). Therefore, to express a functional copy of *nadA*, a construct containing the *nadA-2/3.8* gene (variant 2/3, peptide 8) was transformed into *N. cinerea* ATCC14685 *lpxL1* (see **Supplementary Table S1**), which naturally expresses fHbp_ID100_. The *nadA* construct contains the gene under the control of *nadA-porA* fusion promoter (46) and an upstream erythromycin resistance gene, flanked by regions homologous to the *nadA-4/5* remnant region. mOMVs generated from *N. cinerea* ATCC^®^ 14685™ *lpxL1 nadA-4/5::nadA-2/3.8* containing functional copies of the *fHbp*_ID100_ and *nadA* genes, are referred as OMV-ID100 NadA-8. Antigen expression of fHbp_ID100_ and NadA-8 on the surface of the bacteria were confirmed by flow cytometry (data not shown).

**Figure 1:**
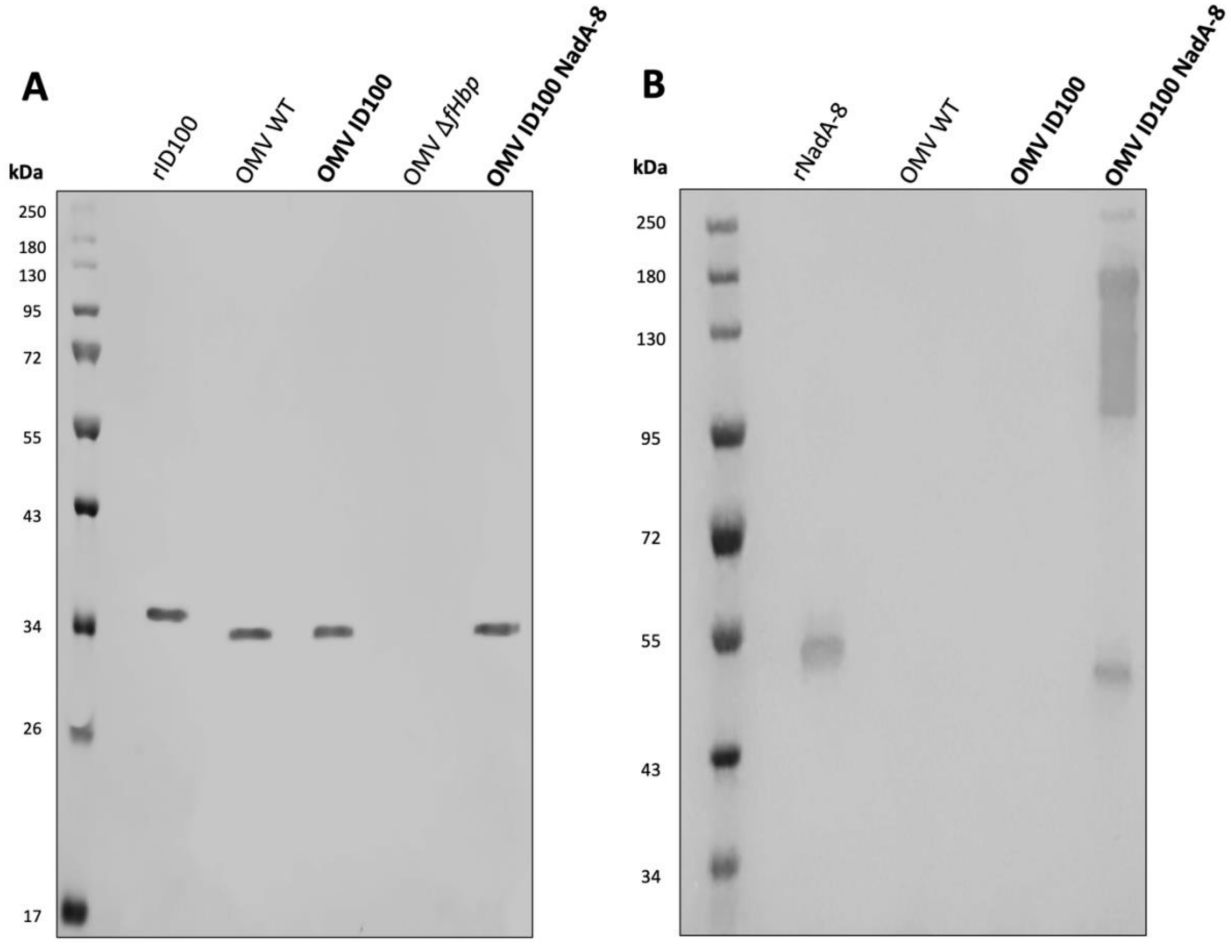
Analysis of the expression of fHbp ID100 and NadA-8 in mOMV. The presence of fHbpID100 in the mOMV from *N. cinerea* strains were analysed by WB with **(A)** JAR5 and the presence of NadA-8 were analysed by WB with **(B)** a polyclonal α-NadA-8. Each lane was loaded with either 2.5 μg of OMVs or 0.1 μg of recombinant proteins. Recombinant fHbp_ID100_ and NadA-8 were used as positive controls.

Following mOMV purification, the expression of fHbp_ID100_ and NadA-8 was determined by WB. As controls for the WB, OMVs were also extracted from *N. cinerea* ATCC^®^ 14685™ WT, *N. cinerea* ATCC^®^ 14685™ *ΔlpxL1* and *N. cinerea* ATCC^®^ 14685™ *ΔfHbp ΔlpxL1*, which are referred as OMV WT, OMV ID100 and OMV *ΔfHbp*, respectively. As shown in **Figure 1A**, the presence of fHbp_ID100_ from OMV-ID100 and OMV-ID100 NadA-8 can be detected by WB. FHbp bands are at the expected molecular weight (MW, ~30 kDa), with the recombinant protein having a higher MW than their counterparts in the mOMV due to the presence of the His-tag.

The expression of NadA-8 from the mOMV was also detected by WB (**Figure 1B**), with multiple bands representing NadA-8 found in OMV ID100 NadA, while the bands were absent in OMV WT and OMV ID100. While the lower MW band represents the monomeric form of NadA-8 (~50 kDa), the higher-MW represents the oligomeric form of the antigen. It has been previously reported that NadA can form oligomers (18).

### Expression of fHbp_ID45_ and NHBA-2 in *N. cinerea* ATCC^®^ 14685™ mOMVs

To express NHBA-2, a construct containing the *nhba-2* gene and an upstream kanamycin resistance gene, flanked by regions homologous to the *lpxL1* gene was transformed into *N. cinerea* ATCC^®^ 14685™ *ΔfHbp*_ID100_ *::fHbp*_ID45_ (see **Supplementary Table S1**). mOMVs generated from *N. cinerea* ATCC^®^ 14685™ *ΔfHbp*_ID100_ *::fHbp*_ID45_ *ΔlpxL1::nhba-*2 containing functional copies of *fHbp*_ID45_ and *nhba-*2 genes, are referred as OMV-ID45 NHBA-2. Antigen expression of fHbp_ID45_ and NHBA-2 on the surface of the bacteria was confirmed by flow cytometry (data not shown).

**Figure 2:**
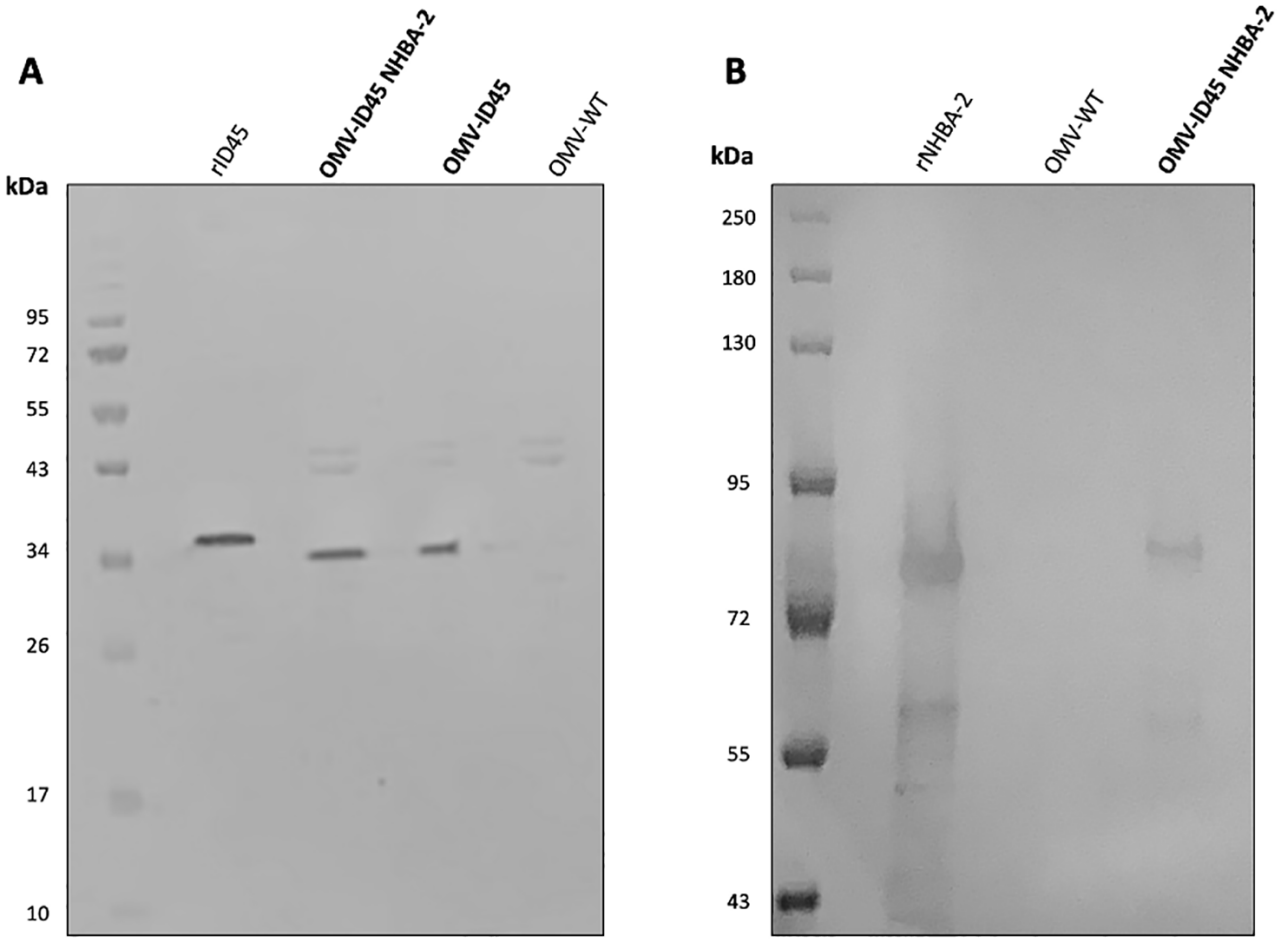
Analysis of the expression of fHbp ID45 and NHBA-2 in mOMV. The presence of fHbp_ID45_ was analysed by WB with **(A)** JAR13 and the presence of NHBA-2 in the mOMV from *N. cinerea* strains was analysed by WB with **(B)** a monoclonal α-NHBA 10C3. Each lane was loaded with either 2.5 μg of OMVs or 0.1 μg of recombinant proteins. Recombinant fHbp_ID45_ and NHBA-2 were used as positive controls.

Following mOMV purification, the expression of fHbp_ID45_ and NHBA-2 was determined by WB. As controls for the WB, OMVs were also extracted from *N. cinerea* ATCC^®^ 14685™ WT and *N. cinerea* ATCC^®^ 14685™ *ΔfHbp*_ID100_ *::fHbp*_ID45_ *ΔlpxL1*, which are referred as OMV WT and OMV ID45, respectively. As shown in **Figure 2A**, the presence of fHbp_ID45_ from OMV-ID45 and OMV-ID45 NHBA-2 can be detected by WB, while it is absent in OMV WT. FHbp bands are at the expected molecular weight (MW, ~30 kDa), with the recombinant protein having a higher MW than their counterparts in the mOMV due to the presence of the His-tag. The expression of NHBA-2 from the mOMV was also detected by WB (**Figure 2B**) while it is absent in OMV WT, with the bands representing NHBA-2 found at the expected MW of ~80 kDa.

### Characterisation of vesicles by DLS and transmission electron microscopy

To assess the vesicle size, mOMV generated in this study were analysed by DLS. The DLS profiles (**Supplementary Figure S4**) indicated a uniform distribution of nanoparticles within the reads consisting of three individual measurements. The nanoparticles were ranging between 81.09 to 98.22 nm (**Table 2**), which falls within the 20-250 nm known range, confirming that the samples contained the expected size of vesicles (47, 48). The average diameter and the polydispersity index (PdI) of each set of mOMV is shown in **Table 2**. All samples displayed low PdI, suggesting near-homogenous mOMVs were extracted.

**Table 2:** Particle size of mOMVs, determined using DLS. PdI refers to polydispersity index.

mOMV	Strain used to extract the mOMV	Z-average diameter (nm)	PdI
OMV-ID45 NHBA-2	*N. cinerea* ATCC^®^ 14685^TM^ *ΔfHbp*_ID100_ *::fHbp*_ID45_ *ΔlpxL1::nhba*-2	81.09	0.196
OMV- ID100 NadA-8	*N. cinerea* ATCC^®^ 14685^TM^ *ΔlpxL1 ΔnadA-4/5::nadA-8*	98.22	0.176

**Figure 3:**
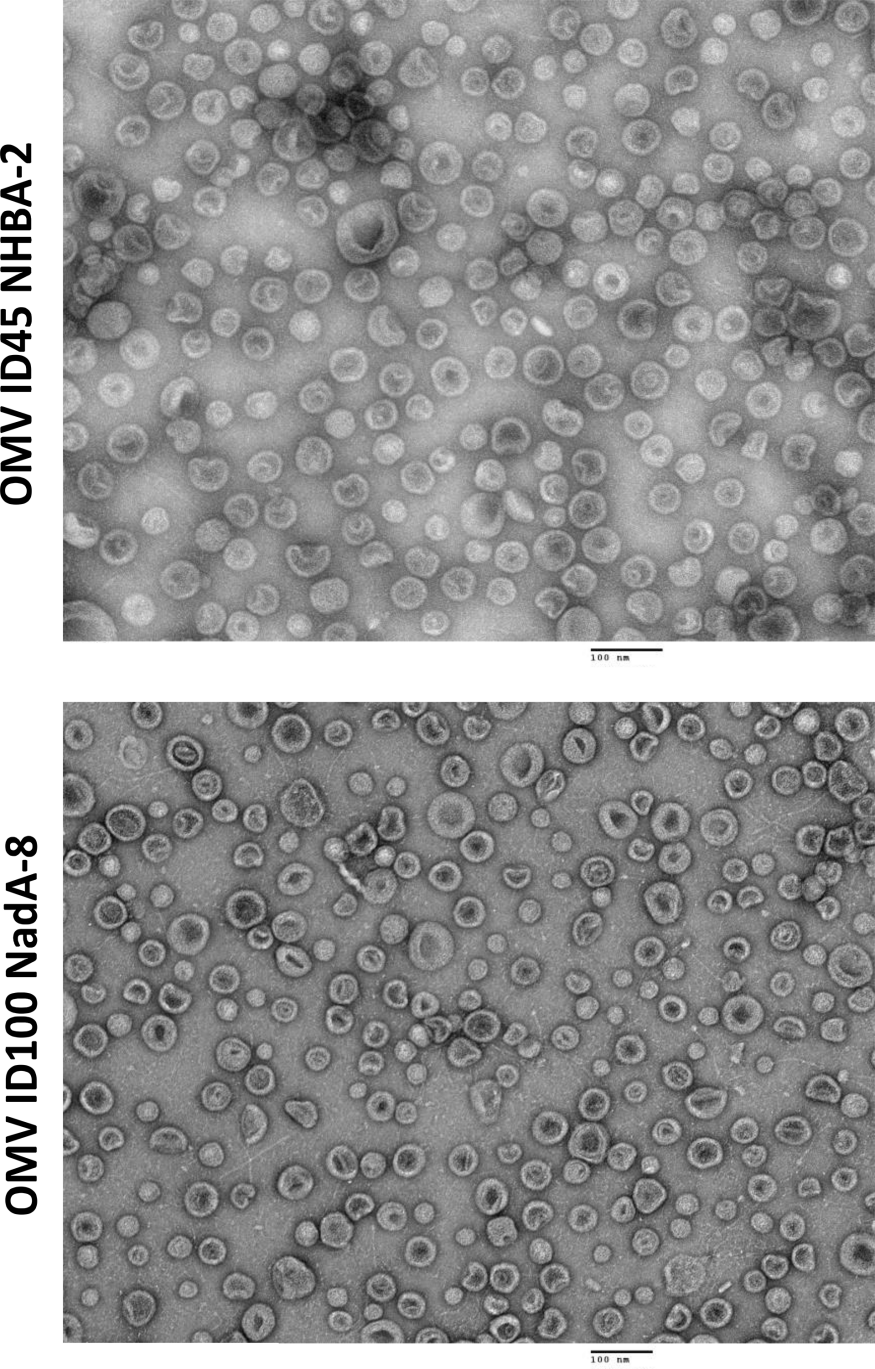
TEM images of mOMVs stained with uranyl acetate. Scale bar = 100 m.

mOMVs from *N. cinerea* ATCC^®^ 14685™ strains carrying different mutations and heterologous antigens were also visualised using TEM, with the OMVs appearing intact and sizes of vesicles being between 100-200 nm (**Figure 3**).

### Confirmation of reduced reactogenicity of mOMVs *in vitro*

**Figure 4:**
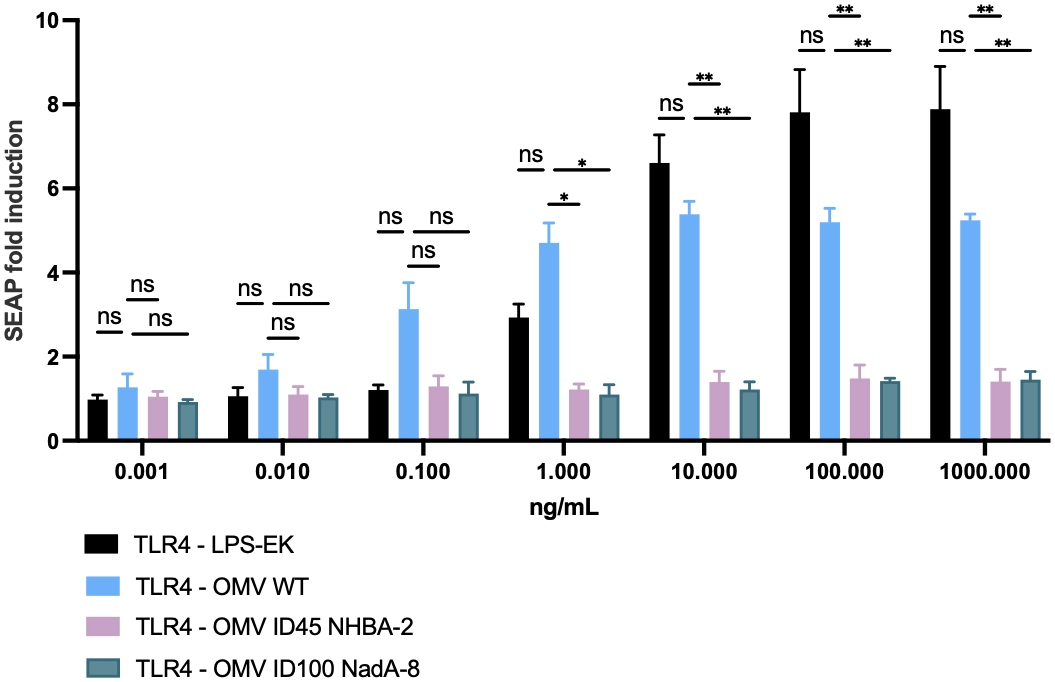
Cellular *in vitro* assay to assess TLR4 activation after stimulation with mOMVs. 2.5 x 10^4^ cells/well of HEK-293 hTLR4, MD2, CD14 were stimulated for 22 hours with 10-fold serial dilutions of mOMV preparations. hTLR4 activation correlates with SEAP induction, measured as per manufacturer’s protocol. Data shown are from three biological replicates, with each replicates containing technical triplicates; data are shown as mean with standard deviation (vertical error bars). LPS-EK: ultrapure lipopolysaccharide from *E. coli* K12; it served as positive control for the assay. HEK-Null cells (which contained the gene reporter system, but lacking hTLR4, MD2 and CD14) were used as a negative control for the assay (data not shown) and showed SEAP fold induction of less than 1.5 when stimulated with OMV or purified LPS-EK throughout the dose range used for the assays. Statistical analysis was performed using Two-Way ANOVA with Dunnett’s multiple comparisons test, where * denotes p-value of <0.05, ** denotes p-value of <0.01, and ns denotes not significant.

To ensure that the *lpxL1* deletion in the mutant strains results in the expected reduction of reactogenicity (49, 50), hTLR4 activation in HEK-Blue hTLR4 cells was studied after stimulation with mOMVs from *ΔlpxL1* and parental *N. cinerea* ATCC^®^ 14685™ strains, to confirm the reduced engagement of TLR4 by LOS. As shown in **Figure 4**, OMVs from *N. cinerea* ATCC^®^ 14685™ WT induced hTLR4 activation in a dose-dependent manner. In comparison, all mOMV preparations from *N. cinerea* ATCC^®^ 14685™ containing the *ΔlpxL1* deletion showed a greatly reduced or no stimulation of hTLR4 (p<0.05 with 10 ng/μL or higher concentrations of mOMVs). Thus, mOMV produced from *N. cinerea* ATCC^®^ 14685™ containing the *ΔlpxL1* deletion showed a reduced hTLR4 activation *in vitro*, irrespective of the different heterologous antigens expressed.

### *In vivo* immunogenicity study of mOMVs expressing fHbp, NadA and NHBA antigens

mOMVs expressing meningococcal antigens were tested in a preclinical murine model to determine whether the *N. cinerea* ATCC^®^ 14685™ mOMVs would induce immune responses against antigens present within the mOMVs. Six groups of 10-15 C57BL/6 mice were immunised with different doses of OMV ID100 NadA-8, OMV ID45 NHBA-2, or with a combination of the two mOMVs or with Alhydrogel adjuvant only (**Table 1**). The dose concentrations used were 10 μg and 20 μg. Mice received three IP injections of each preparation on days 0, 21 and 49. To assess immunogenicity of the mOMVs, serum samples were tested by ELISAs against the same type of mOMVs used to immunise the mice or against recombinant proteins.

**Figure 5:**
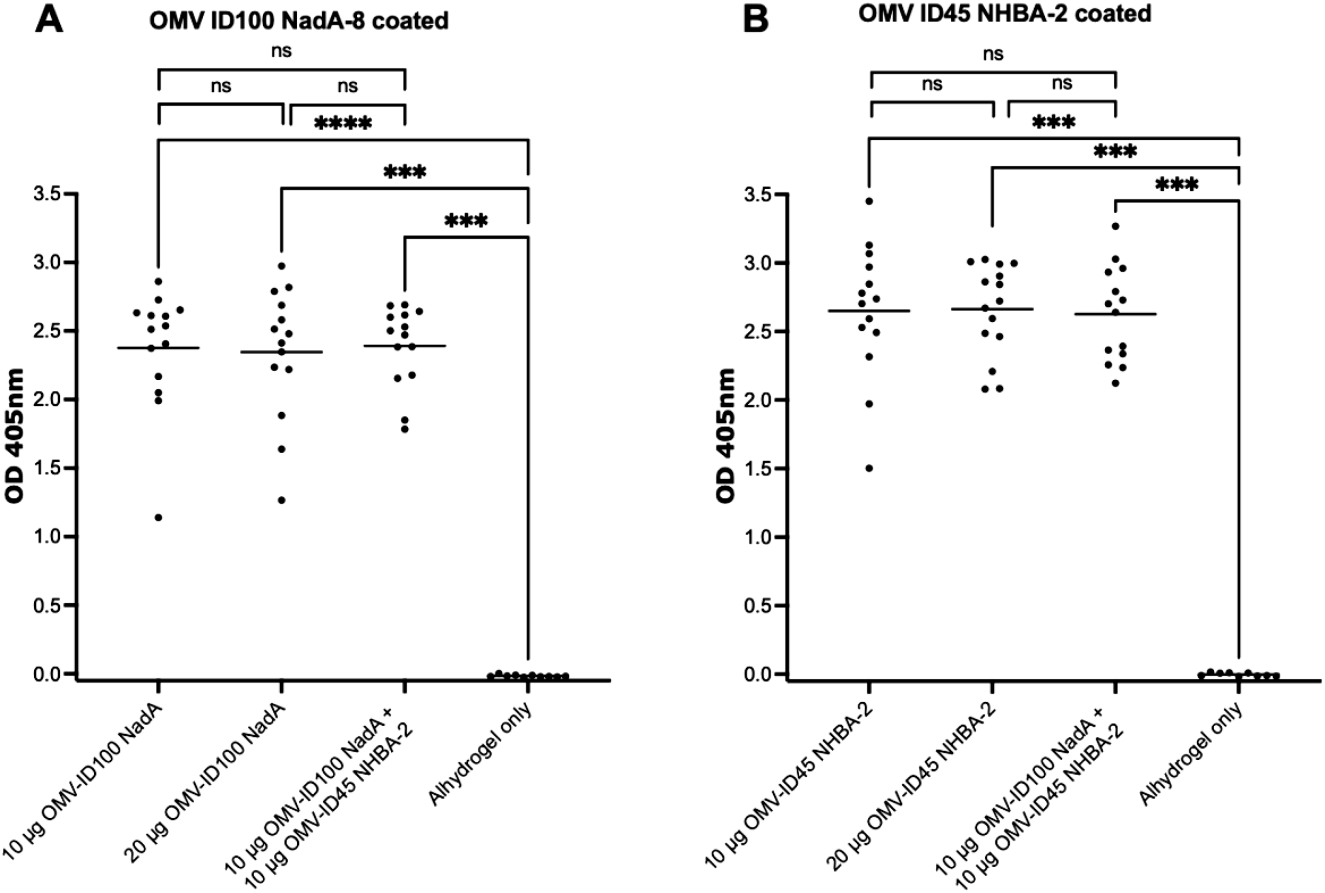
Sera responses of mice immunized with OMV-ID100 NadA-8 and/or OMV-ID45 NHBA-2, when plates were coated with their respective mOMVs. IgG antibody levels measured in sera diluted at 1:27,000 when plates were coated with A) OMV-ID100 NadA and B) OMV-ID45 NHBA-2. Statistical analysis was performed using Kruskal-Wallis test with Dunn’s multiple comparison post-test, where ns denotes not significant, *** denotes p-value of <0.001 and **** denotes p-value of <0.0001.

Immunisation with the various mOMV preparations at different dose concentrations induced a high level of IgG (high OD_405_ values at a 1:27,000 dilution) against their respective mOMVs (corresponding to the same mOMVs that were used to dose each group) (**Figures 5A, B**). The mean OD_405_ ranged from 2.34-2.39 on plates coated with OMV ID100 NadA-8, and 2.62-2.66 for plates coated with OMV ID45 NHBA-2. Immunisation with Alhydrogel only yielded low OD_405_ values of <0.05 (**Figures 5A, B**).

**Figure 6:**
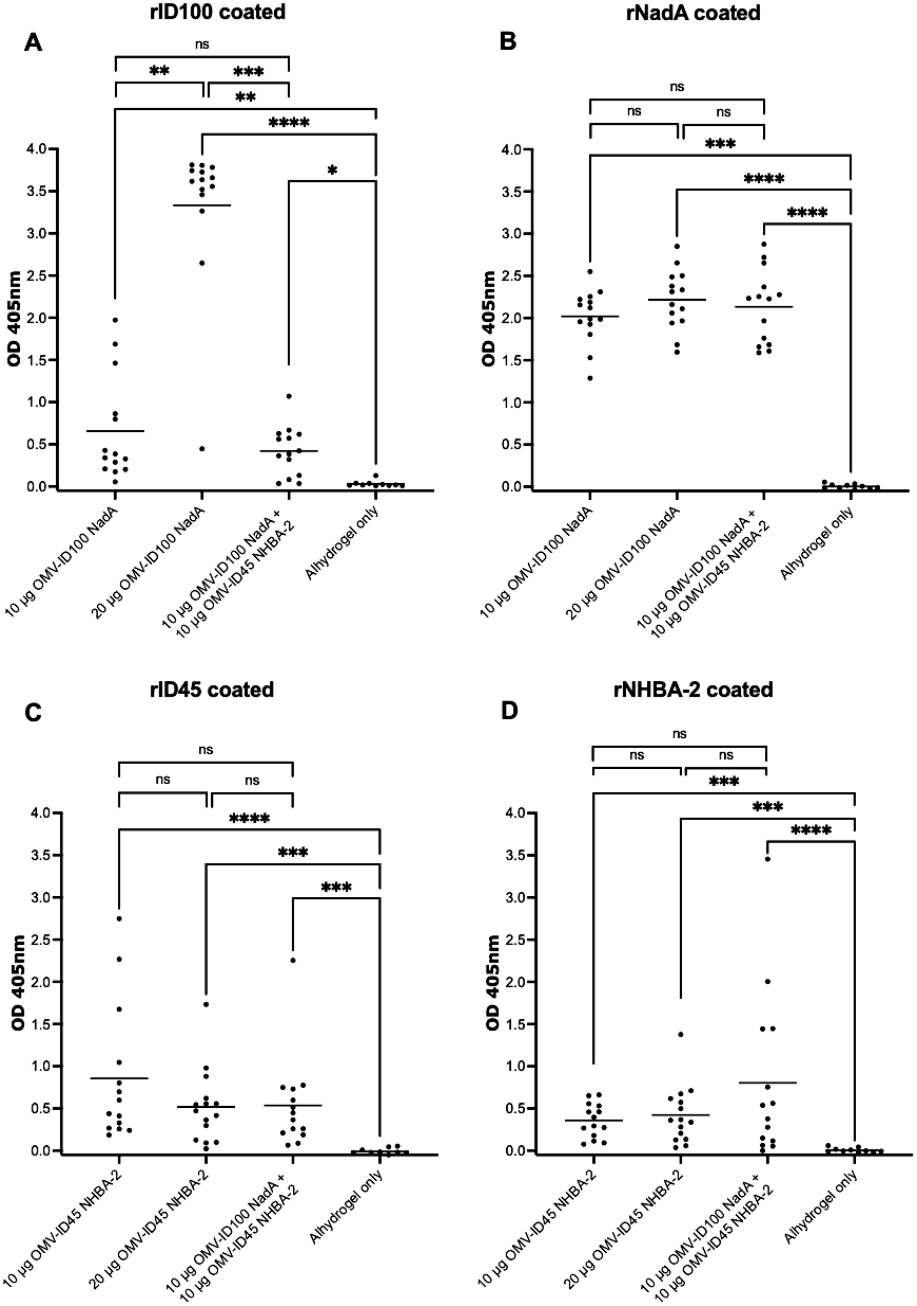
Sera responses of mice immunized with OMV-ID100 NadA-8 and/or OMV-ID45 NHBA-2, when plates were coated with recombinant proteins. **(A)** IgG antibody levels measured in sera diluted at 1:80 when plates were coated with rID100 protein. **(B)** IgG antibody levels measured in sera diluted at 1:4,000 when plates were coated with rNadA-8 protein. **(C)** IgG antibody levels measured in sera diluted at 1:40 when plates were coated with rID45 protein. **(D)** IgG antibody levels measured in sera diluted at 1:40 when plates were coated with rNHBA-2 protein. Line denotes the mean of IgG antibody levels. Statistical analysis was performed using Kruskal-Wallis test with Dunn’s multiple comparison post-test, where ns denotes not significant, * denotes p-value of <0.05, ** denotes p-value of <0.01, *** denotes p-value of <0.001 and **** denotes p-value of <0.0001.

For ELISA plates coated with recombinant proteins, the OD_405_ at 1:40 serum dilution is shown for plates coated with rID45 and rNHBA-2, 1:80 serum dilution is shown for plates coated with rID100 and 1:4,000 serum dilution is shown for plates coated with rNadA, as these dilutions are within the linear part of the OD_405_ curve. Sera from mice immunised with OMV-ID100 NadA-8 yielded significantly higher OD_405_ readings towards rID100 compared to sera from mice injected with Alhydrogel only (**Figure 6A**). The mean OD_405_ is statistically higher at 20 μg compared to 10 μg of mOMVs. (**Figure 6A**). Immunisation with OMV ID100 NadA-8 induced a significantly higher levels of antibodies against rNadA-8 compared to mice immunised with Alhydrogel only (**Figure 6B**) with no statistical difference observed between the dose of 10 μg and 20 μg (**Figure 6B**).

Sera from animals immunised with OMV-ID45 NHBA-2 yielded significantly higher OD_405_ values against rID45 and rNHBA-2 compared to sera from animals injected with Alhydrogel only (**Figures 6C, D**). No significant difference was observed between the dose of 10 μg compared to 20 μg (**Figures 6C, D**).

### Serum antibodies cross-react with proteins from representative MenB and commensal *Neisseria* strains

To examine the cross-reactivity of mOMV-induced serum antibodies against MenB, WB were performed against whole cell lysates of MenB MC58. Whole cell lysates of commensal strains *N. cinerea* ATCC^®^ 14685™, *Neisseria sicca* ATCC^®^ 29256™ and *N. sicca* ATCC^®^ 29259™ were also used, as these strains are phylogenetically placed near *N. meningitidis* (45, 51, 52).

**Figure 7:**
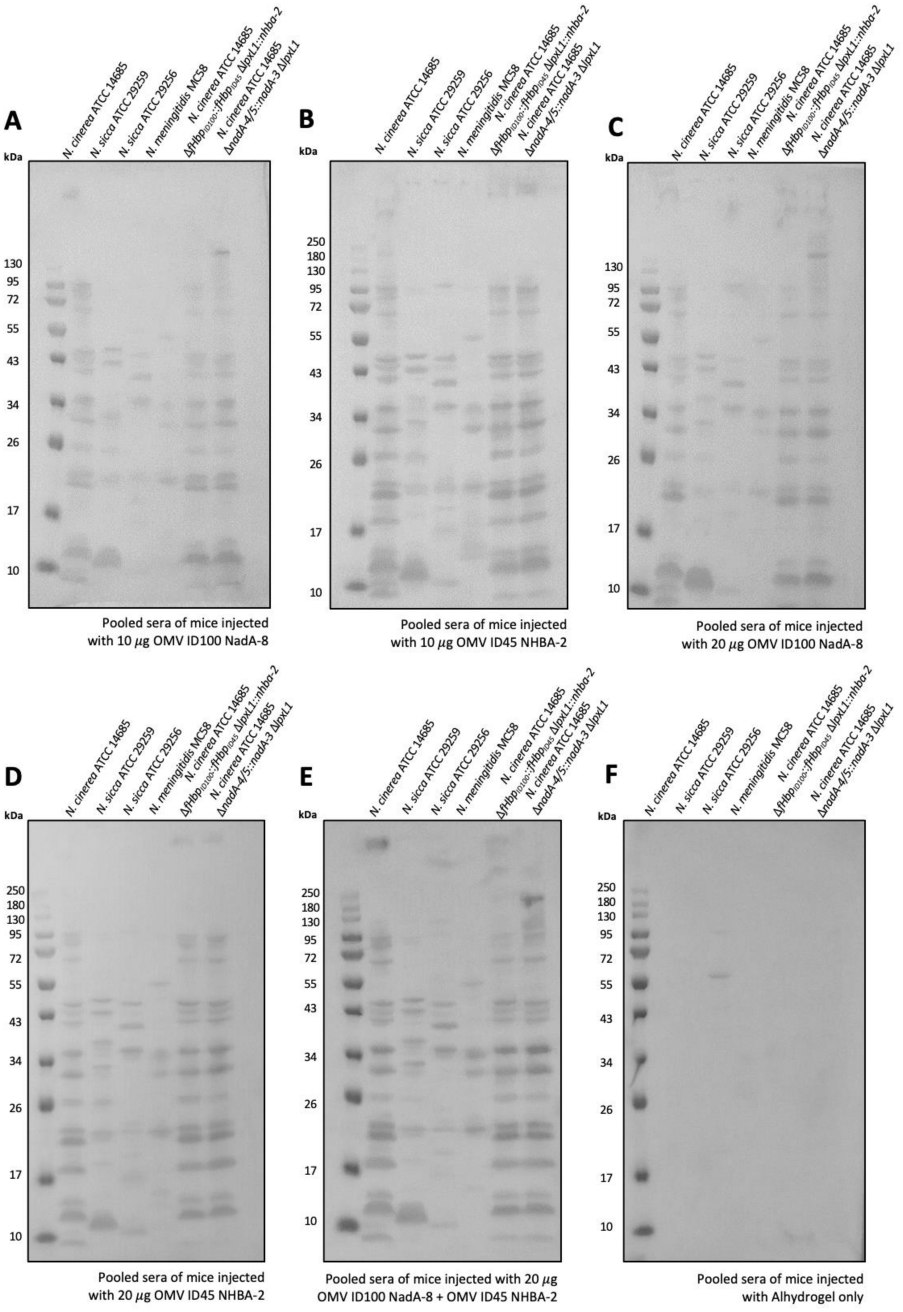
Sera antibodies induced by immunization with mOMVs expressing meningococcal antigens cross-react with MenB. Sera from mice immunized with **(A)** 10 μg OMV-ID100 NadA-8 **(B)** 10 μg OMV-ID45 NHBA-2, with **(C)** 20 μg OMV-ID100 NadA-NadA-8 **(D)** 20 g OMV-ID45 NHBA-2 **(E)** 10 μg OMV-ID100 NadA-8 + 10 μg OMV-ID45 NHBA-2 and **(F)** Alhydrogel only were used to probe whole-cell lysates of MenB MC58, as well as *N. cinerea* ATCC^®^ 14685^TM^ and *N. sicca* ATCC^®^ 29259^TM^.

Pooled sera from mice immunized with mOMVs (OMV ID100 NadA-8 and/or OMV ID45 NHBA-2) recognised prominent bands in *N. meningitidis* MenB MC58 of apparent MW of ~55 kDa, 34 kDa and 20 kDa (**Figure 7**). The sera were also cross-reacting against commensal *Neisseria* lysates, including *N. cinerea* ATCC^®^ 14685™, from which the mOMVs were extracted from. In comparison, pooled sera from mice immunised with Alhydrogel only did not display any reactivity to any *Neisseria* strains (**Figure 7**).

### mOMV induced strong serum bactericidal activity against a NadA-expressing MenB strain

The functional activity of the mouse sera was investigated by serum bactericidal activity (SBA) assays. With studies identifying that antibodies to NHBA alone lack bactericidal activity (53–55), we have selected MenB strains that express either NadA and/or variants of fHbp. SBA was performed against *N. meningitidis* serogroup B strains 5/99 (Mar 07) and M17 240102, with strain 5/99 (Mar 07) carrying fHbp from subfamily A and NadA from variant 3, and strain M17 240102 carrying fHbp from subfamily B.

Pooled sera from mice immunised with OMV ID100 NadA elicited bactericidal activity against MenB strain 5/99 (Mar07) with a titre of 32,768; pooled sera from mice immunised with Alhydrogel only had SBA titre of <8 (**Table 3**). No difference in SBA titres was observed between the sera of mice immunised with 10 μg and 20 μg of mOMVs. In addition, sera from mice dosed with 10 μg of OMV ID45 NHBA-2 displayed a titre of 64, while sera from mice dosed with 20 g of OMV ID45 NHBA-2 displayed a titre of <8 (**Table 3**). A combination of OMV ID100 NadA and OMV ID45 NHBA-2 at a total protein concentration of 20 μg for each dose also elicited bactericidal activity, with a titre of 16,384 (**Table 3**).

**Table 3:** SBA titres against MenB strains after three doses of mOMVs.

Groups	Dose concentration (μg)	*N. meningitidis* serogroup B strains	
		5/99 fHbp subfamily A (ID23) NadA variant 2/3 NHBA peptide 20	M17 240102 fHbp subfamily B (ID13) NHBA peptide 17
OMV ID100 NadA	10	32,768	<8
OMV ID100 NadA	20	32,768	<8
OMV ID45 NHBA-2	10	64	<8
OMV ID45 NHBA-2	20	<8	<8
OMV ID100 NadA + OMV ID45 NHBA-2	10+10	16,384	<8
Alhydrogel only	0	<8	<8

It should be noted that the lower limit of quantification for the SBA due to the specified stating dilution is 8. If any samples have SBA titres below 8, this will be reported as <8.

Against strain M17 240102, which expresses fHbp from subfamily B and does not contain a functional *nadA* gene, pooled sera from all the groups did not display any bactericidal activity (**Table 3**). This suggests that although OMV ID100 NadA expresses fHbp from subfamily B, pooled sera from mice immunised with OMV ID100 NadA could not elicit bactericidal activity against *N. meningitidis* expressing fHbp subfamily B.

## Discussion

To address the need for an affordable MenB vaccine, we conducted a study to evaluate the design and immunogenicity of two mOMVs from commensal *N. cinerea* using a mouse model. This study aimed to investigate the possibility to deliver multiple *Neisseria* antigens to the immune system in a two vesicle mOMV preparation. We demonstrated that the use of mOMVs (OMV ID100 NadA and OMV ID45 NHBA-2) from commensal *N. cinerea* induces immune responses *in vivo* against total OMV preparations and against individual bacterial antigens that are expressed in the mOMV after recombinant cloning of their genetic sequences.

In this study, we have expressed variants of fHbp and NHBA-2 in *N. cinerea* mOMVs, which are antigens that are present in all MenB strains (12, 18). We have additionally inserted the *nadA-8* gene in *N. cinerea* ATCC^®^ 14685™ *ΔlpxL1*. Although *nadA* is absent in some virulent strains and most non-virulent strains of *N. meningitidis*, the gene is generally associated with hypervirulent strains and found in approximately 75% of hypervirulent MenB strains (18, 20, 21). NadA-8 has previously been highlighted to be potently immunogenic (56), which led to its incorporation in the multicomponent Bexsero vaccine (57).

*N. cinerea* provides an attractive parental species to produce a vaccine candidate due to being a BSL-1 bacteria (58). All mOMVs were proven to possess the correct antigen profiles by WB. Toxicity studies were conducted to measure the *in vitro* response by hTLR4 to the presence of *Neisseria* mOMVs. It was demonstrated that mutants with the *lpxL1* gene deletion could produce mOMVs that lowered stimulatory potential of hTLR4. This consistently shows that this modification detoxifies the LOS compared to OMV WT and prevent activation of downstream innate proinflammatory pathways such as transcription factor NF-κB activation.

Parenteral administration of mOMVs from *N. cinerea* ATCC^®^ 14685™ expressing at least two meningococcal antigens induced immune responses in mice against total mOMV preparations and against individual bacterial antigens present in the mOMVs, in a three-dose regimen. The potency of immune responses varied between the different mOMVs used in the mice experiment. In particular, while NadA and fHbp_ID100_ induced high levels of serum antibody responses, fHbp_ID45_ and NHBA-2 induced low levels of serum antibody responses, as shown by the OD values in ELISAs. The reason for this could be due to expression levels of the antigens on the outer membrane. Future work will include measuring the levels of antigen expression and optimizing the expression of the antigens by investigating different promoter sequences as well as exploring the effect of the deletion of immunodominant OMPs such as PorB. Deletion of PorA, PorB and/or RmpM has been shown to be achievable in *N. meningitidis* and the mOMVs enhanced the cross-protective effect of OMV vaccine (59).

Sera from mice dosed with mOMVs were able to recognise multiple proteins on all the bacterial lysates tested, ranging in sizes from 10 kDa to 130 kDa. As expected, these groups of sera recognised proteins from the parental strain (*N. cinerea* wild-type) but also several proteins from the meningococcal strain MC58, showing a level of cross-reactivity with the pathogenic strain used as the antigenic basis of fHbp and NHBA variants in the 4CMenB vaccine.

The levels of antibody recognition in ELISAs were later paralleled by the potency of sera in SBA. SBAs tested against *N. meningitidis* 5/99 (Mar07), which expresses fHbp subfamily A and NadA, demonstrated very high bactericidal activity by antibodies from mice dosed with OMV ID100 NadA with a titre of 32,768, while a titre of 64 or below was observed with OMV ID45 NHBA depending on the concentration. A combination of OMV ID100 NadA and OMV ID45 NHBA-2 at a total protein concentration of 20 μg led to a titre of 16,384. In contrast, SBAs tested against *N. meningitidis* M17 240102 which expresses fHbp subfamily B displayed a titre of <8 in all groups, including sera from animals immunised with OMV-ID100 NadA. It is possible that higher levels of IgG against fHbp_ID100_ are required to elicit bactericidal activity against test strains expressing these antigens. Future work will therefore involve improving the expression levels of fHbp and NHBA on mOMVs by using alternative promoters, testing alternative adjuvants and broadening the number of MenB strains to be evaluated in SBA assays.

Although NHBA-2 alone may not stimulate high IgG levels, its presence on vesicles may still serve a purpose at enhancing the immunogenicity of fHbp in a synergistic pattern. In previous studies, mice were injected with either a formulation of recombinant fHbp_ID1_ or NHBA-2 and obtained sera to act as a source of IgG in bactericidal studies. Treatment against a meningococcal strain with a high amino acid similarity for both antigens, alongside human serum complement source, resulted in far higher bactericidal killing when incubated with 1:1 preparation of both mouse groups, compared to treatment of just one of these sera groups (15, 60).

In conclusion, our study indicates that it is possible to deliver multiple *Neisseria* antigens using mOMV combinations and to elicit immune responses against these antigens. We also showed that NadA is highly immunogenic and vaccines containing this antigen induce sera with high SBA against NadA-expressing meningococcal isolates. Other *Neisseria* antigens can be presented in combination, and induce serum antibody responses and SBA. Further optimization is needed to enhance the functional activity of the immune sera against all variants of fHbp and to broaden strain coverage.

## Data Availability

The original contributions presented in the study are included in the article/supplementary material. Further inquiries can be directed to the corresponding author.
