# Supplementary material for: Characterisation and Immunogenicity of Neisseria cinerea outer membrane vesicles displaying NadA, NHBA and fHbp from Neisseria meningitidis serogroup B
Source: Front Immunol. 2024 Sep 24;15:1473064. doi: 10.3389/fimmu.2024.1473064 (PMC11458423; doi:10.3389/fimmu.2024.1473064)
Supplement: Supplementary file 1 [file DataSheet1.pdf]

Characterisation and Immunogenicity of *Neisseria cinerea* outer membrane vesicles displaying NadA, NHBA and fHbp from *Neisseria meningitidis* serogroup B

Shathviga Manoharan*, Theo A. Farman, Stavroula Piliou, Pietro Mastroeni

Department of Veterinary Medicine, University of Cambridge, Cambridge CB3 0ES, UK

*Corresponding author (sm2690@cam.ac.uk)

SUPPLEMENTARY MATERIALS

SUPPLEMENTARY TABLES

Supplementary Table S1: Bacterial strains used in this study.

Strains	Characterisation	Source/Reference
N. cinerea ATCC® 14685™	Wild-type containing a functional copy of *fHbp* (ID100)	American Type Culture Collection (ATCC), Manassas, VA
N. cinerea ATCC® 14685™ Δ*lpxL1*	The *lpxL1* gene was replaced with the kanamycin resistance cassette	(Piliou et al., 2023)
N. cinerea ATCC® 14685™ Δ*lpxL1* Δ*fHbp*$_{ID100}$	The *lpxL1* gene was replaced with the kanamycin resistance cassette and the native *fHbp*$_{ID100}$ gene was replaced with the erythromycin resistance cassette	(Piliou et al., 2023)
N. cinerea ATCC® 14685™ Δ*fHbp*$_{ID100}$*::fHbp*$_{ID45}$ Δ*lpxL1*	The *lpxL1* gene was replaced with the kanamycin resistance cassette. The native *fHbp* gene was deleted and replaced with the *fHbp* ID45 gene (under the control of *nadA-porA* fusion promoter) and the chloramphenicol resistance cassette	(Piliou et al., 2023)
N. cinerea ATCC® 14685™ Δ*fHbp*$_{ID100}$*::fHbp*$_{ID45}$ Δ*lpxL1::nhba-2*	The native *fHbp* gene was deleted and replaced with the *fHbp* ID45 gene (under the control of *nadA-porA* fusion promoter) and the chloramphenicol resistance cassette. The *lpxL1* gene was replaced with the *nhba-2* gene (under the control of *cat* promoter) and the kanamycin resistance cassette.	This study
N. cinerea ATCC® 14685™ Δ*lpxL1* *nadA-4/5::nadA-2/3.8*	The *lpxL1* gene was replaced with the kanamycin resistance cassette. The native *nadA-4/5* locus was deleted and replaced with the *nadA-2/3.8* gene (*nadA* peptide 8 under the control of *nadA-porA* fusion promoter) and the erythromycin resistance cassette	This study

Supplementary Table S2: Plasmids obtained from Genscript.

Plasmids	Characteristics
Δ*lpxL1::nhba*-2_pUC18	The *nhba-2* gene with the chloramphenicol (*cat)* promoter and an upstream kanamycin resistance cassette, flanked by regions homologous to the *lpxL1* gene (with DUS), cloned into pUC18
Δ*nadA-4/5::nadA-2/3.8*(erm^R)_pUC18	The *nadA-2/3* gene with the *nadA-porA* fusion promoter and an upstream erythromycin resistance cassette, flanked by regions homologous to the *nadA-4/5* locus (with DUS), cloned into pUC18

[1]The *nadA-porA* fusion promoter is a modified *porA* promoter, in which the polyguanine tract between -35 and -10 sites has been replaced with the corresponding sequence from the *nadA* gene (Pajon et al., 2013).

Supplementary Table S3: List of primers used in this study. F = forward, R = reverse, Up = upstream, Do = downstream, int = internal and ext = external. Primers were synthesised and supplied by Sigma-Aldrich.

Primer name	Sequence (5'-3')	Description
pUC18_F	CAGGTCGACTCTAGAGGATCCC	To amplify the *nadA* and *nhba-2* constructs from the plasmids obtained from Genscript
pUC18_R	CAGCTATGACCATGATTACGAATTCG	
nadA-4/5_ext_F	TGACCGACGAACACAAACGCA	External primers to check the deletion of nadA-4/5 and the insertion of Δ*nadA-4/5::nadA-2/3*
nadA-4/5_ext_R	TGGAACGCGCGATTGATGTAA	
*ermB*_int_R	CAGTTTCGTCGTTAAATGCCC	Reverse internal primer to *ermB*, to confirm mutants by PCR. It works with nadA-4/5_ext_F to confirm Δ*nadA-4/5::nadA-2/3*
lpxL1_ext_F	GACTTGCGTCCAAACTC	To anneal upstream of the recombination site of the *lpxL1* gene.
kan_int_R	CGGCCAGATCGTTATTCAGTAAG	Reverse internal to *kanR*, to confirm mutants by PCR. It works with lpxL1_ext_F to confirm Δ*lpxL1::nhba-2*

Supplementary Table S4: Summary of the antibodies and their concentrations used for Western blots (WB) and enzyme-linked immunosorbent assay (ELISA).

Antibodies	Concentration for WB	Concentration for ELISAs	Source
Monoclonal mouse JAR 5	1:500*	1:1,000*	NIBSC (13/216)
Monoclonal mouse JAR 13	2 μg/mL	0.1 μg/mL	Merk (MABF2667)
Polyclonal rabbit α-NHBA-2	0.5 μg/mL	0.1 μg/mL	Genscript
Polyclonal rabbit α-NadA-8	0.5 μg/mL	0.1 μg/mL	Genscript
Monoclonal human α-NHBA 12E1	10 μg/mL	-	Creative Biolabs
Alkaline phosphatase conjugated α-mouse IgG	1:3,000*	1:3,000*	Sigma (A9316)
Alkaline phosphatase conjugated α-rabbit IgG	1:3,000*	1:3,000*	Sigma (A3687)
Alkaline phosphatase conjugated α-human IgG	1:20,000*	-	Sigma (A1543)

*From manufacturer's stock (see code numbers in Source column).

SUPPLEMENTARY RESULTS

Analysis of the *nadA* locus in *N. cinerea* ATCC 14685

The *nadA* gene was previously reported to be present in two *N. cinerea* strains, and in strain ATCC® 14685™, the gene was found at the border of two distinct contigs (Muzzi et al., 2013). However, the integrity of the gene was not established. Homologue of the *nadA* gene is found in contigs 20.1 (NCBI accession number: NZ_ACDY02000021.1) and 17.1 (NCBI accession number: NZ_ACDY02000018.1). Two genes at the end of contig 20.1 (locus tags NEICINOT_05139 and NEICINOT_05140) and one gene at the beginning of contig 17.1 (locus tag NEICINOT_05054) showed a relatively low percentage identity to *nadA* from *N. meningitidis* MC58. The homology of NEICINOT_05139 and NEICINOT_05140 was limited to the N-terminal portion of NadA, while the homology of NEICINOT_05054 was limited to a C-terminal portion of NadA that is common to a family of YadA-like surface proteins.

The NEICINOT_05139 gene is flanked at the 5' end with a putative ribosome-binding site, -10 and -35 binding sites, as well as nine TAAA repeats (**Supplementary Figure S1**). Because of the presence of the TAAA repeats, the gene may undergo phase variation, even though the repeats are not in the coding region. The NEICINOT_05054 gene is flanked at the 3' end by a putative terminator sequence, determined by ARNold (http://rssf.i2bc.paris-saclay.fr/toolbox/arnold/). Interestingly, a terminator sequence was also predicted within the NEICINOT_05140 coding region, potentially abruptly terminating the transcription of the gene.

To determine whether these borders of the contigs are found adjacent to each other in the chromosome, the end of contig 20.1 and the beginning of contig 17.1 was amplified by PCR. As shown in **Supplementary Figure S2**, the expected size band of ~3 kb was observed, signifying that the contigs are placed adjacent to each other within the chromosome.

While three annotated genes show some homology to the *nadA* gene present in *N. meningitidis*, the functionality of those genes has not been established (see **Supplementary Figure 3**).

SUPPLEMENTARY FIGURES

AAAAAATGCCGTCTGAACATTCAGACGGCACCCCATATCCTGACAAAATTAAGAAACGACACCGGCAG

TATTGGCGGCAGCATAACATGCACATGTTAACAGATATCAATACCGAACCACCCGCCGGCAAGAATCA

AATAAATAAATAAATAAATAAATAAATAAATAAATAAATTACGGCAAAGTATTGTATATATGCCTCCC

TTTCACATATACTTCAATATGTAAGCAAACTTGGCGGGAATGAAAATACTTGCAAAAGATTTTCGCCC

CGTTTTTCATCCACTCACAAAGGTAATGAGCC**ATG**AAACACTT

Supplementary Figure S1: Nucleotide sequence of the upstream region of NEICINOT_05139 in *N. cinerea* ATCC 14685. Putative -35 and -10 domains and a possible ribosomal binding site are indicated with an underline. The translation start codon is shown in bold. The repeats of the tetranucleotide TAAA are highlighted in black. This upstream region has an 89% nucleotide sequence identity to the MenB MC58 *nadA* promoter region.

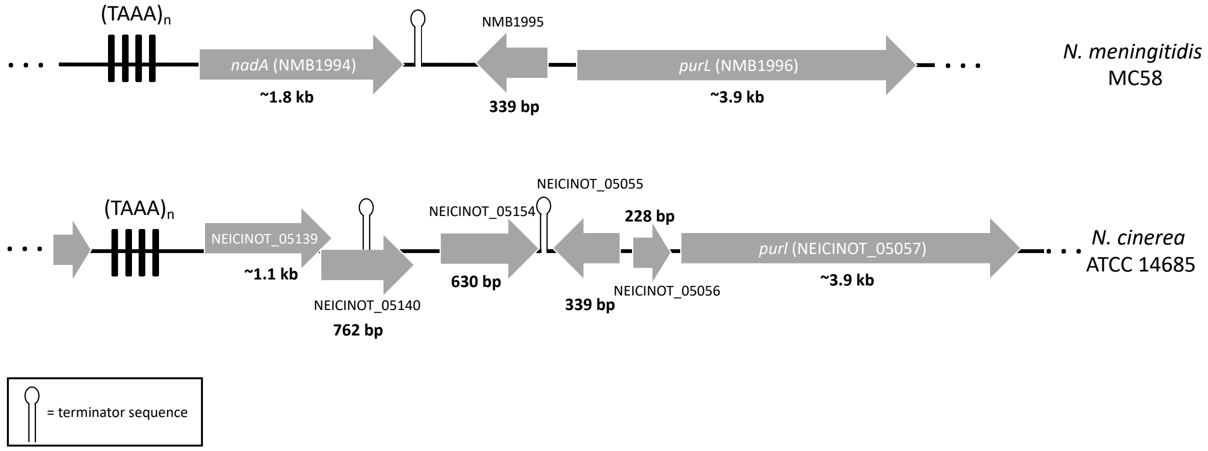

Supplementary Figure S2: Schematic comparison of the genetic arrangement of the *nadA* locus in *N. meningitidis* MC58 (serogroup B) and *N. cinerea* ATCC 14685.

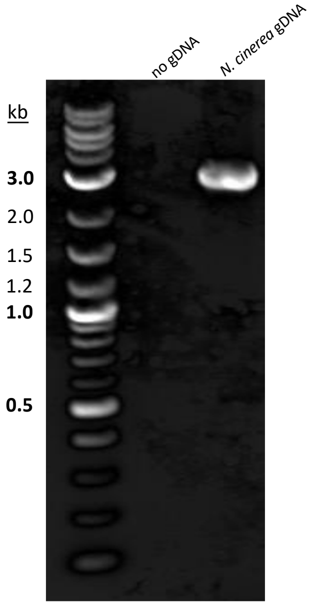

Supplementary Figure S3: PCR product of *nadA* region amplified in *N. cinerea* ATCC14685. The *nadA* region of *N. cinerea* ATCC 14685, which is found in the border of two contigs, were amplified by PCR.

A

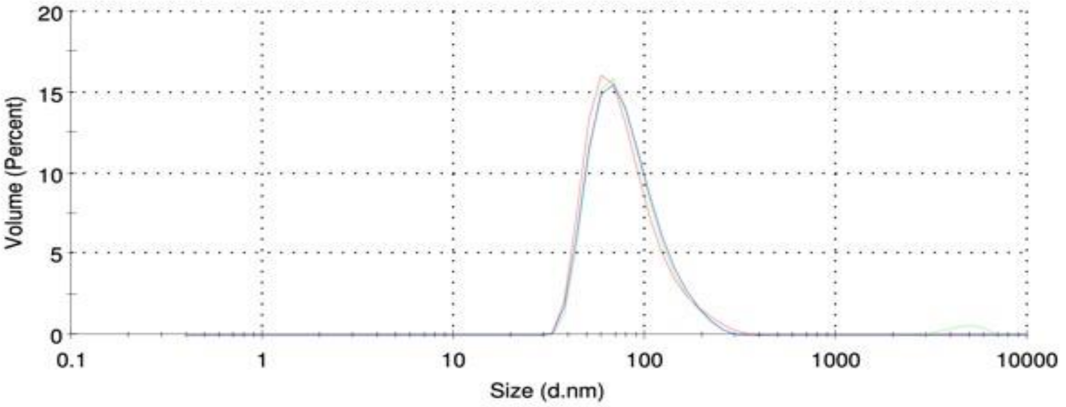

B

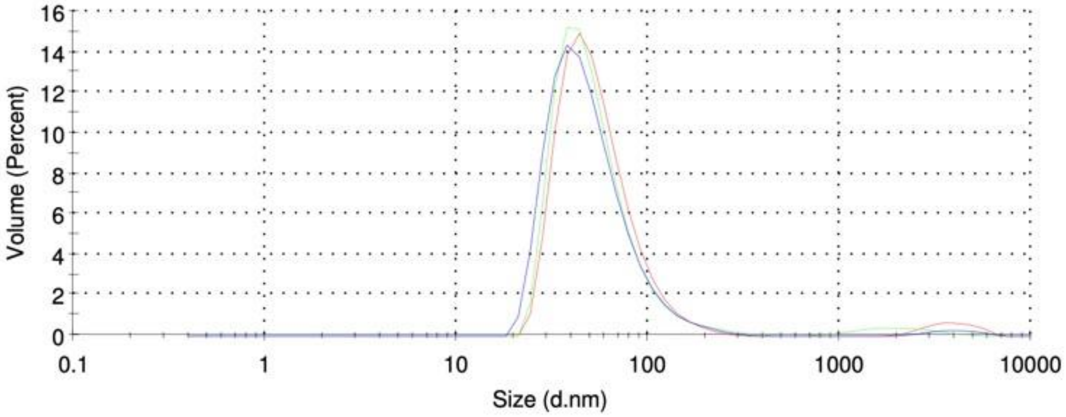

Supplementary Figure S4: The vesicle size distribution of **(A)** OMV ID100 NadA-8 and **(B)** OMV ID45 NHBA-2**,** using dynamic light scattering. Each line represents a repeat of measuring the size of the mOMVs.

REFERENCE

Muzzi, C., Mora, A., Pizza, M., et al. (2013) Conservation of meningococcal antigens in the genus Neisseria. *mBio*, 4 (3): 163–176. doi:10.1128/mBio.00163.

Pajon, R., Fergus, A.M. and Granoff, D.M. (2013) Mutant Native Outer Membrane Vesicles Combined with a Serogroup A Polysaccharide Conjugate Vaccine for Prevention of Meningococcal Epidemics in Africa. *PLoS ONE*, 8 (6): e66536. doi:10.1371/journal.pone.0066536.

Piliou, S., Farman, T.A., Marini, A., et al. (2023) Commensal Neisseria cinerea outer membrane vesicles as a platform for the delivery of meningococcal and gonococcal antigens to the immune system. *Vaccine*, 41 (52): 7671–7681. doi:10.1016/j.vaccine.2023.11.034.
